# Approaches to overcome flow cytometry limitations in the analysis of cells from veterinary relevant species

**DOI:** 10.1186/s12917-020-02299-2

**Published:** 2020-03-06

**Authors:** Julia Hunka, John T. Riley, Gudrun F. Debes

**Affiliations:** 1 Department of Microbiology and Immunology, Sidney Kimmel Medical College and Sidney Kimmel Cancer Center, Thomas Jefferson University, 233 S 10th Street, Philadelphia, PA 19107 USA; 2 Faculty of Veterinary Medicine, Ludwig-Maximilians-Universität, Munich, Germany

**Keywords:** Flow cytometry, Multiparameter, Veterinary species

## Abstract

**Background:**

Flow cytometry is a powerful tool for the multiparameter analysis of leukocyte subsets on the single cell level. Recent advances have greatly increased the number of fluorochrome-labeled antibodies in flow cytometry. In particular, an increase in available fluorochromes with distinct excitation and emission spectra combined with novel multicolor flow cytometers with several lasers have enhanced the generation of multidimensional expression data for leukocytes and other cell types. However, these advances have mainly benefited the analysis of human or mouse cell samples given the lack of reagents for most animal species. The flow cytometric analysis of important veterinary, agricultural, wildlife, and other animal species is still hampered by several technical limitations, even though animal species other than the mouse can serve as more accurate models of specific human physiology and diseases.

**Results:**

Here we present time-tested approaches that our laboratory regularly uses in the multiparameter flow cytometric analysis of ovine leukocytes. The discussed approaches will be applicable to the analysis of cells from most animal species and include direct modification of antibodies by covalent conjugation or Fc-directed labeling (Zenon™ technology), labeled secondary antibodies and other second step reagents, labeled receptor ligands, and antibodies with species cross-reactivity.

**Conclusions:**

Using refined technical approaches, the number of parameters analyzed by flow cytometry per cell sample can be greatly increased, enabling multidimensional analysis of rare samples and giving critical insight into veterinary and other less commonly analyzed species. By maximizing information from each cell sample, multicolor flow cytometry can reduce the required number of animals used in a study.

## Background

Fluorescence-activated cell sorting (FACS) and flow cytometry have been essential immunological tools since the invention of FACS in the late 1960s [1–3], as they enable identification, characterization, and isolation of defined leukocyte subsets [4, 5]. Flow cytometry employs fluorochrome-labeled antibodies that detect cell surface or intracellular antigens [6, 7], a method that was first developed for characterization of cells and tissues by microscopy [8]. Recent advances in the development of novel fluorochromes and instrumentation (i.e. flow cytometry analyzers and sorters) allow for the theoretical analysis of up to 50 parameters in a single staining panel [9], and a 28-color panel has recently been demonstrated [10]. Polychromatic experiments enable the simultaneous measurement of a larger number of cell surface and intracellular markers, thereby facilitating the analysis of infrequent cell subsets or limited cell samples [5, 11–13]. Therefore, many institutions have acquired high capacity flow cytometers, and the analysis of > 10 fluorochromes has become routine in the study of human and mouse cells.

The house mouse (*Mus musculus*) is the most frequently used species in biomedical research and, as a consequence, a large spectrum of reagents and genetic models are available [14, 15].. However, animal species other than the house mouse may represent more suitable models of specific human physiology, disease or anatomy, and can also enable studies of comparative medicine and/or of zoonotic pathogens in their natural hosts [14, 16, 17]. An example is the guinea pig, which has been a model for human infectious diseases for 200 years, and enabled disease research and vaccine development in tuberculosis [18, 19]. More recent examples for the use of non-mouse species in biomedical research include pigs and sheep in orthopedics and Alzheimer’s disease [20–22] and dogs in oncology [23].

Flow cytometry is a key method in immunological studies [24] encompassing biomedical, veterinary, agricultural, and wildlife research, but the method is also routinely employed in veterinary clinical laboratories [25, 26]. Unfortunately, we face many limitations in the analysis of non-mouse animal samples, including lower availability of commercially or otherwise available antibodies to cell antigens and reduced options for fluorochrome labels by commercial antibody suppliers. It is also not uncommon to receive limited amounts of hybridoma supernatant rather than purified antibody. In addition, antibodies for non-standard species tend to be more expensive. Due to this absolute and relative lack of reagents, the design of state-of-the-art multicolor flow cytometry staining panels is much more difficult than it is for mouse or human cell samples.

Our laboratory studies lymphocyte recirculation using lymph vessel cannulation in sheep, which was pioneered by Bede Morris [27, 28]. Due to a number of limitations, afferent lymph vessels cannot be readily cannulated in mice or humans, and lymph vessel cannulation in sheep allows for the analysis of lymphocytes during their physiological recirculation through tissues [29–31]. Here we present technical approaches that are commonly employed by our laboratory to increase the number of parameters analyzed by flow cytometry per cell sample from sheep [32–36]. The discussed approaches are compatible with the analysis of cells from most other animal species and include direct modification of antibodies by covalent conjugation or Fc-directed labeling (Zenon™ labeling kits), labeled secondary antibodies and other second step reagents, labeled receptor ligands and species cross-reactivity. Detailed guidelines for the use of flow cytometry, including general protocols, are extensively discussed elsewhere [11, 24].

## Results

### Selection of fluorochromes

**Fig. 1:**
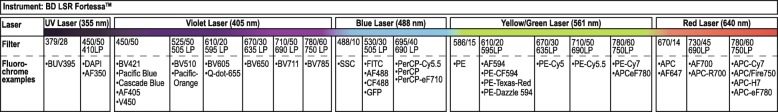
Flow cytometry laser and fluorochrome chart used for our studies. The BD LSR Fortessa™ used in this study is depicted with our laser and corresponding filter set-up, as well as fluorochrome examples commonly used in our laboratory. Abbreviations: AF™, Alexa Fluor™; APC, allophycocyanin; BUV, Brilliant UltraViolet™; BV, Brilliant Violet™; CF, Cyanine-based fluorescent dye; Cy, Cyanine; DAPI, 4′,6-diamidino-2-phenylindole; eF, eFluor; FITC: fluorescein isothiocyanate; LP, long pass; PE, R-phycoerythrin; PE-Cy5, phycoerythrin-cyanine5 conjugate; PE-Cy7, phycoerythrin-cyanine7 conjugate; PerCP, peridinin chlorophyll-A protein

When designing multicolor staining panels for flow cytometry, one is limited to the use of fluorochromes compatible with available flow cytometers. Therefore, the technical specificities of the available instrumentation determine the fluorochromes in a staining panel. For the panels presented in this paper we used the BD LSR Fortessa™ cell analyzer. Our machine has 5 lasers, UV (355 nm), violet (405 nm), blue (488 nm), yellow/green (561 nm), and red (640 nm), and can simultaneously detect up to 18 colors plus forward- (FSC) and side scatter (SSC) properties (Fig. 1). Figure 1 depicts the specific laser and filter set-up of our flow cytometer, its theoretically available colors, as well as examples for fluorochromes commonly used in our laboratory. As advised [37], we aim to choose fluorochromes with minimal spectral overlap, and online resources such as the Spectrum Viewer from BD, Fluorescence Spectra Viewer from Thermo Fisher Scientific, or the BioLegend Spectra Analyzer help with assessing the degree of spectral overlap and potential spillover. The simultaneous use of fluorochromes with extensive spectral overlap is more feasible with appropriate compensation, carefully titrated antibodies, and when the antibodies recognize distinct cell populations, e.g. B cells vs. T cells; the approach is less suitable for co-expression studies [24, 38]. However, each specific staining panel will need to be tested and optimized on available instrumentation. More details on optimal fluorochrome combinations and discussions on appropriate compensation techniques are described elsewhere [12, 24, 37].

When designing staining panels, we select brighter fluorochromes for antibodies that bind rare antigens as advised [37]. Companies such as BioLegend provide a relative brightness index for fluorochromes but also warn that the brightness can vary depending on the antibody, antigen, or cell type, and that it is also influenced by instrumentation. Consequently, titrating the antibody is always recommended. Examples of brighter fluorochromes that we used in our panels of this paper include phycoerythrin (PE), Alexa Fluor™ (AF™) 594, phycoerythrin-cyanine 7 (PE-Cy7), allophycocyanin (APC), and Brilliant Violet™ 421 (BV421) (Fig. 1). In an ideal scenario, monoclonal antibodies for each cell antigen are available in all possible fluorochromes. However, even for human antigens this is not the case and is further from reality in veterinary species. Thus, initial antibody staining panel design will depend on easily available and previously validated antibodies (“what is in the refrigerator”) and approaches to expand the panel.

### Covalent labeling of antibodies and species-cross-reactive antibodies

**Fig. 2:**
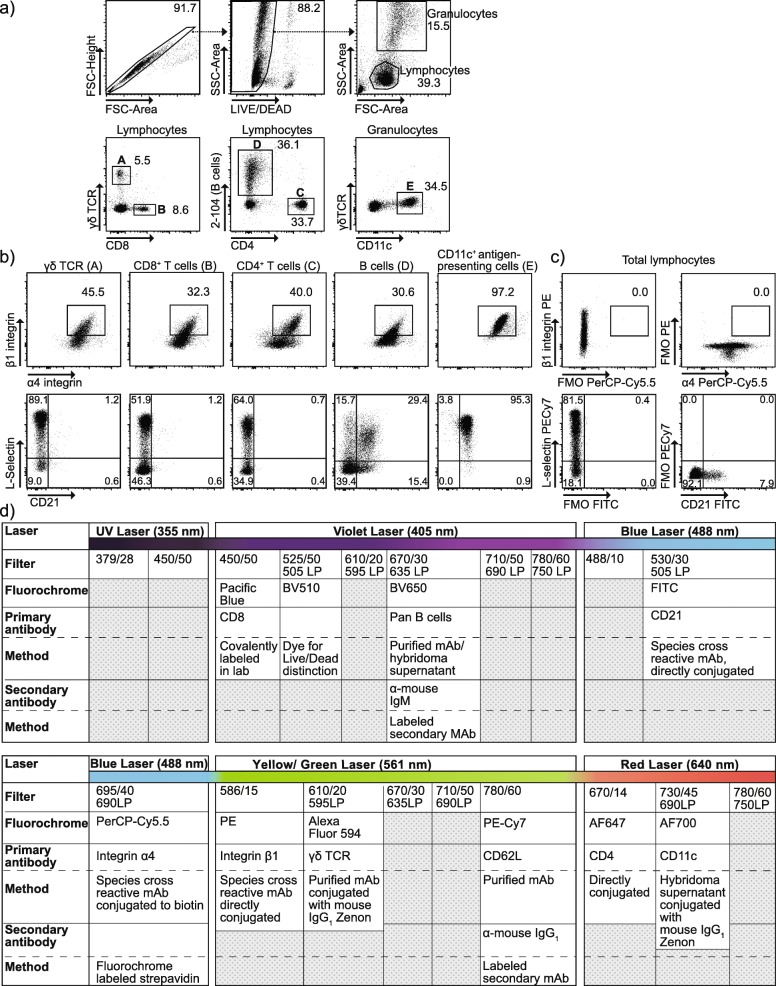
Staining and gating strategy to identify leukocyte populations in ovine blood. Peripheral blood mononuclear cells were obtained by density gradient centrifugation and (**a**) cells were gated on FSC-Height and FSC-Area to exclude doublets. Singlets were further gated on SSC-Area and LIVE/DEAD™-dye^low^ cells to exclude dead cells. Viable cells were then gated for lymphocytes and granulocytes based on FSC and SSC properties. Lymphocytes were gated for γδ T cells (**a**), CD8^+^ T cells (**b**), CD4^+^ T cells (**c**), and B cells (**d**). Granulocytes were gated for CD11c^+^ antigen presenting cells (E). (**b**) Each leukocyte subset (A-E) was analyzed for expression of α4- and β1-integrins (top row) and CD21 and CD62L (bottom). (**c**) Fluorescence-minus-one controls (FMO) for fluorochromes used to stain α4- and β1-integrins, CD21, and L-selectin in (**b**) using the total lymphocyte gate shown in Panel (**a**). (**d**) Table indicating the reagents and flow cytometer configuration used in this staining panel, including the respective fluorochrome, as well as the methods employed to visualize each ovine cell surface marker. Reagents that were obtained as covalently labeled reagents are marked as “directly conjugated”. (a and b) Numbers within dot plots represent percentages. Abbreviations: AF™, Alexa Fluor™; Bio, biotin; BV, Brilliant Violet™; FITC, fluorescein isothiocyanate; LP, long pass; mAb, Monoclonal antibody; PE, R-phycoerythrin; PE-Cy7, phycoerythrin-cyanine7 conjugate; PerCP-Cy5.5, peridinin chlorophyll-A protein-cyanine; SA, streptavidin

Antibody vendors have a variable supply, and antibodies for veterinary species are generally available in a limited number of fluorochrome labels and are often unconjugated. BioRad, for example, has a sound variety of anti-ovine and other veterinary antibodies, most of which are only available purified or conjugated to fluorescein isothiocyanate (FITC) or PE. A method to broaden the fluorochrome range is to label purified antibodies by using a reactive labeling kit that covalently binds fluorochromes to reactive protein groups, such as amines. In contrast to purified antibodies, non-purified antibodies (i.e. hybridoma supernatants, ascites fluid) cannot be labeled covalently without also labeling other protein components in the fluid, but they can be selectively labeled with the Zenon™ labeling method (discussed below). Covalent labeling kits for variable amounts of antibody are available for numerous fluorochromes from commercial vendors, such as Invitrogen™ by Thermo Fisher Scientific, Novus Biologicals, Abcam, or Biotium™. The labeling procedure follows simple protocols provided by the manufacturer and takes less than 3 hours. The newly labeled antibody is immediately ready for staining but should be titrated and validated prior to use in an experiment. The reactive label works for all animal species and IgG subclasses, and the covalently labeled antibody is stable and can be stored for usage over several months. For example, we labeled purified anti-ovine CD8 with a Pacific Blue™ antibody labeling kit and included it in a multicolor panel to detect CD8^+^ T cells among sheep blood lymphocytes (Fig. 2a). Within the same staining panel, lymphocytes were additionally gated for γδ T cells (Population A), CD4 T cells (C), B cells (D), and CD11c^+^ antigen presenting cells (E) distinguished within the high side scatter granulocyte population (Fig. 2a).

To broaden the antibody repertoire and fluorochrome spectrum, many laboratories use antibodies that are raised against antigens in one species but exhibit documented cross-reactivity for a different species. When antibodies are produced for use in mice and humans, they are more likely to be commercially available in a larger variety of colors. In our example panel, we took advantage of documented cross-reactivity of anti-human α4- and β1-integrin and anti-bovine CD21 antibodies with their corresponding sheep molecules [39, 40] and analyzed all cell subsets (A-E) for these markers, as well as CD62L (Fig. 2b).

Staining controls are particularly important for multiparameter analyses [37]. In a fluorescence minus one (FMO) control all antibodies of the panel, except for one, are included in their respective fluorochromes, allowing assessment of spectral overlap into the “empty” channel (Fig. 2c). Figure 2d depicts the individual fluorochromes and antibodies to antigens used in the staining panel of Fig. 2 and lists the method by which the individual staining was achieved.

### Zenon™ labeling kits

**Fig. 3:**
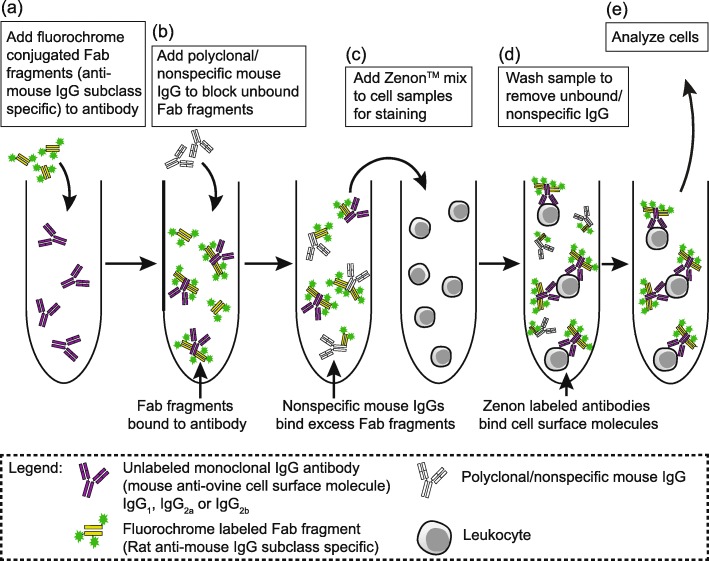
The principle of Zenon™ antibody labeling. To label mouse monoclonal IgG antibodies, we employed Zenon™ technology. All steps were performed at room temperature. (**a**) The unlabeled target monoclonal mouse IgG (purple) is mixed with mouse IgG subclass-specific fluorochrome-conjugated Fab fragments (green/yellow) from the corresponding Zenon™ kit. The fluorochrome-conjugated Fab fragments bind the target antibody. (**b**) Addition of nonspecific polyclonal mouse immunoglobulin (white) blocks excess unbound Fab fragments. (**c**) The mix of newly Zenon™-labeled antibodies and blocked excess Fab fragments is added to the cell sample and binds to its respective cell surface antigens. Antibodies (Zenon-labeled or directly conjugated) to other cell surface molecules can be included in this step. (**d**) Excess antibody and blocked Fab fragments are washed away, and (**e**) the stained cells are ready to be analyzed or fixed

An additional approach to overcome limited fluorochrome availability for domesticated and other animal species is the use of Zenon™ labeling kits (Invitrogen™, Thermo Fisher Scientific) [33, 34, 41, 42]. The Zenon™ labeling technique uses fluorochrome-labeled Fab antibody fragments that recognize the IgG subclass (Fc portion) of its target antibody (Fig. 3). This noncovalent conjunction enables the labeling of human, mouse and rabbit antibodies, which can be purified antibodies, hybridoma culture supernatant, or ascites fluid [43]. Therefore, the Fab fragment of the Zenon™ kit has to recognize the species (human, mouse or rabbit) and the IgG subclass (IgG_1_, IgG_2a_ or IgG_2b_) of the specific target antibody. To label the target antibody, it is mixed with the fluorochrome-labeled Fab fragments of the Zenon™ kit, and the mixture is incubated for 5 min (Fig. 3a). Next, the Zenon™ blocking reagent is added to the mixture and incubated for another 5 min (Fig. 3b). The Zenon™ blocking reagent is a nonspecific immunoglobulin mix from the same species as the target antibody and will bind to excess fluorochrome-labeled Fab fragments (Fig. 3b). Finally, together with other antibodies of the staining panel, the Zenon™ mixture is added to the cell sample for staining (Fig. 3c). After washing to remove unbound nonspecific immunoglobulins, the cells are ready to be analyzed by flow cytometry (Fig. 3e).

For the labeling of purified monoclonal antibodies, our laboratory follows the Zenon™ labeling protocol provided with the kit, which recommends the use of 1 μg of target antibody, 5 μl of the Zenon™ labeling complex and 5 μl of the Zenon™ block. For some very bright fluorochromes and antibodies that recognize highly expressed cell surface antigens, such as anti-γδ TCR antibody labeled with Zenon™ AF™594 (Fig. 2), we only use 2.5 μl of the Zenon™ labeling Fab fragment and 2.5 μl of the Zenon™ blocking reagent to label 1 μg of antibody. However, for antibody hybridoma supernatants of unknown concentration we found the use of 10 μl supernatant and 5 μl of each the Zenon™ labeling complex and Zenon™ block works best in most cases and may be determined by titration for each batch of supernatant. Figure 2 shows an example of antibody supernatant labeling to stain CD11c^+^ antigen presenting cells.

One option that the Zenon™ protocol provides is to stop the labeling after the first step (before adding the blocking reagent) for storage at 4 °C. While our laboratory sometimes stores the Zenon labeled antibody for 1–2 h in the refrigerator, the manufacturer’s protocol allows for storage of up to several weeks with the addition of 2 mM sodium azide (Invitrogen™). The use of the Zenon™ labeling kits is quick and allows flexibility in staining because the fluorochrome can easily be changed by using the isotype-specific Zenon™ kit in a different color. Multiple Zenon™ labeled antibodies can be used simultaneously in the same staining panel, and isotype controls can be labeled in the same manner as the staining antibodies. Each new antibody-Zenon combination should be titrated to determine the best dilution.

### Labeled secondary antibodies and other second step reagents

For the flow cytometric analysis of cells from veterinary species fluorochrome-labeled secondary antibodies are widely used. Monoclonal and polyclonal secondary antibodies are produced in a diverse array of host species and are commercially available in a broad range of colors. The use of monoclonal secondary (and primary) antibodies is preferred as they usually achieve more consistent staining with less background. Secondary antibodies are employed to bind primary antibodies (which recognize antigens on target cells) without cross-recognition of target species antigens. Therefore, secondary antibodies recognize the species and isotype of the primary antibody and are adsorbed to or otherwise unreactive with antigens on cells of the target species. For example, a monoclonal mouse IgM antibody that recognizes sheep B cells (clone 2–104) [44] was visualized with a BV650-conjugated rat anti-mouse IgM monoclonal antibody (Fig. 2a and d). In the same staining panel, mouse anti-ovine CD62L clone DU1–29 was detected with a PE-Cy7-conjugated rat anti-mouse IgG_1_ monoclonal antibody (Fig. 2b and d). Thus, use of secondary antibodies leads to signal amplification and is versatile, allowing for the selection of less commonly used fluorochromes and easy matching to a variety of panels.

**Fig. 4:**
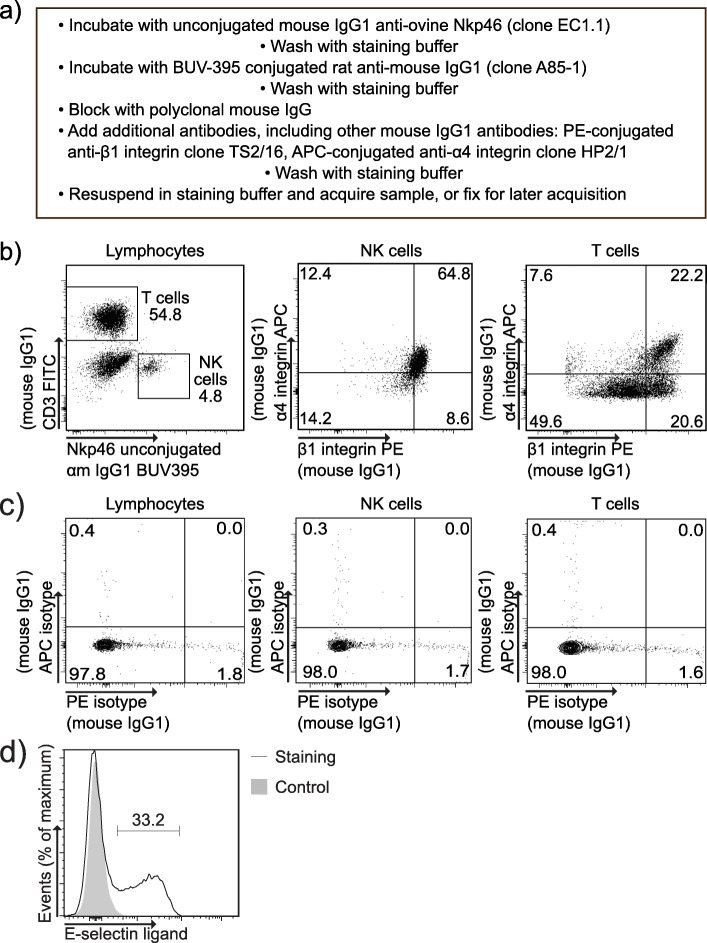
Secondary antibody staining with multiple same-isotype primary antibodies and cell-surface molecule detection by ligand binding. (**a**) Summary of the steps to stain with an isotype-specific secondary antibody when its target isotype antibody is present multiple times in the same staining panel. (**b**) Staining of NKp46 with an isotype-specific secondary antibody (anti-mouse IgG_1_) in the presence of anti α4- and β1-integrin antibodies, which are of the same isotype as the anti NKp46 antibody. Peripheral blood mononuclear cells were pre-gated on single live lymphocytes as in Fig. 2a and CD3^+^ T cells and CD3^−^NKp46^+^ NK cells analyzed for expression of α4- and β1-integrins. (**c**) Corresponding isotype control staining for α4- and β1-integrins. (**d**) E-selectin ligand expression on CD4^+^ T cells from afferent lymph of adult sheep was determined by flow cytometry using an E-selectin-human IgG fusion protein. Cells were pre-gated as in Fig. 2a. As a negative control, staining was performed in buffer containing EDTA. (**b**-**d**) One representative of five individually analyzed sheep is shown. Abbreviations: αm, anti-mouse; APC, allophycocyanin; BUV, Brilliant ultra violet; FITC, fluorescein isothiocyanate; PE, R-phycoerythrin

As a general rule, it is possible to use multiple secondary antibodies in the same staining panel as long as they recognize different immunoglobulin classes or IgG subclasses (e.g. anti-mouse IgG and anti-mouse IgM as shown in Fig. 2, or anti-mouse IgG_1_, and anti-mouse IgG_2a_). Even when using a single secondary antibody, the isotypes of all antibodies in the staining panel must be considered and the primary antibody that is targeted should be the only one recognized by the secondary antibody. However, including two additional staining steps allows for detection of an unlabeled primary antibody in the presence of additional (labeled) antibodies of the same isotype without cross-recognition by the secondary antibody. The first step includes only the primary antibody, followed by only the isotype-specific secondary antibody. After a requisite blocking step with excess unlabeled antibody of the isotype of the target antibody, additional antibodies of the same isotype can be used in a third staining step. Using this approach our laboratory visualized CD62L expressing cells (Fig. 2) as well as natural killer (NK) cells (Fig. 4a and b) with anti-IgG1 secondary antibodies in the presence of other IgG1 staining antibodies. In Fig. 4, unlabeled EC1.1, a monoclonal mouse IgG_1_ that recognizes the ovine natural cytotoxicity receptor NKp46 [45] was visualized by rat anti-mouse IgG_1_, clone A85–1, conjugated to BUV395 (Fig. 4a and b). Following the secondary labeling, the cells were blocked with non-specific polyclonal mouse IgG to saturate any free valences of the secondary antibody, rendering it unable to interfere with the other mouse IgG_1_ antibodies in the staining panel. Finally, mouse IgG_1_ antibodies against α4- and β1-integrins were employed in the staining procedure (Fig. 4a and b). Isotype control antibodies of the same species, antibody isotype and fluorescent label can be incorporated in the same manner as the staining antibodies (Fig. 4c).

Biotinylated antibodies also increase the fluorochrome spectrum and signal in flow cytometry. Streptavidin is commercially available in many different fluorochromes and binds biotin on the primary antibody with high affinity. This leads to signal amplification, making it particularly useful for detection of antigens with low density per cell, and stressing the importance to titrate both the biotinylated primary antibody and the conjugated streptavidin. Many antibodies are commercially available in biotinylated format and purified antibodies can be biotinylated using antibody/protein biotinylation kits or Zenon™ technology (see above).

### Labeled receptor ligands

When antibodies for cell surface receptors are unavailable or when ligand binding ability, as opposed to simple receptor expression is the aim of the study, labeled ligands can be used in flow cytometry. Employing this method, we have previously analyzed ovine lymphocytes for expression of costimulatory molecules B7.1/B7.2 and skin homing marker E-selectin ligand (epitopes that include cutaneous lymphocyte antigen) by evaluating binding of CTLA4-human IgG and mouse E-selectin-human IgG_1_ chimeric proteins, respectively [32–34]. Here, we show an example in which E-selectin binding was visualized with an APC-conjugated mouse monoclonal antibody that recognizes the human IgG_1_ portion of the E-selectin chimeric protein (Fig. 4d). After gating lymph-borne CD4 T cells (as in Fig. 2a), we analyzed their percentage of E-selectin ligand expression (Fig. 4d). Because E-selectin binding requires calcium, a control staining was performed in EDTA-containing buffer (Fig. 4d). Alternative controls will depend on the specific ligand used in an experiment, and examples include staining with irrelevant IgG fusion proteins or blockade of staining with excess unlabeled ligand.

## Discussion

In this methods paper, we present several approaches to overcome flow cytometry limitations in the analysis of veterinary species. During our studies we faced several technical issues. For example, certain antibody clones were not sufficiently labeled by direct covalent labeling kits. This is an old problem and known causes of labeling resistance include: buffer components react with the dye, suboptimal pH, or reactive amine groups lie within the antigen-binding site of the antibody [46]. Zenon antibodies can be a solution for labeling monoclonal IgG antibodies that do not label well with labeling kits. For example, our ovine CD4 monoclonal antibody (44.38) did not yield satisfying staining quality when conjugated with the Invitrogen Pacific Blue antibody labeling kit. However, using the Zenon technology yielded superior results. Another common difficulty is the conjugation of IgM antibodies because most labeling kits raise pH and denature the pentameric structure of IgM [47]. Some manufacturers, such as Thermo Fisher Scientific, offer specific protocols optimized for IgM labeling. In the case of the (mouse IgG1) anti-NKp46 clone, neither covalent conjugation nor Zenon™ technology were effective methods for labeling, and we had to employ the staining method outlined in Fig. 4a. Unfortunately, not all commercial antibody suppliers have consistent quality controls in place and we have occasionally seen commercially labeled antibodies that are unreliable. We also found that in one case the Zenon™ mix interfered with the staining of a different antibody in the same staining panel. Specifically, the anti-ovine B cell antibody (2–104) is a mouse IgM and its detection by an anti-mouse IgM secondary antibody was blunted. An ELISA revealed that the Zenon™ blocking reagent that is included in the kits contained both mouse IgG and mouse IgM, and the latter was competing with our mouse IgM primary antibody for binding by the secondary anti-mouse IgM antibody. We solved the issue by using purified IgG for blocking rather than the Zenon™ kit blocking component.

Certain tandem dyes are sensitive to degradation, leading to a weaker signal and detection in other fluorochrome channels. For example, PE-tandem dyes are susceptible to degradation by handling, storage, and light [48, 49]. We also found that the tandem dye PE-Cy7, is sensitive to extended fixation with PFA, which can degrade the fluorophore and lead to artifactual strong signals in the PE channel. We found that the following precautions prevent tandem conjugate degradation: staining at 4 °C in the dark, careful and extensive washing after fixation (i.e. twice with sufficient buffer volume), and storage at 4 °C in the dark for no longer than 24 h. Another potential issue, which we have not encountered in our studies, is the interference of Brilliant Violet and other ultra-bright antibodies with each other when used in the same panel. Such issues can be addressed by using specialized staining buffers. BD Bioscience, for example, offers a specific buffer for staining with Brilliant™ dyes [50].

While we present several simple approaches to broaden the number of flow cytometric parameters per cells, additional approaches exist that we have not utilized so far. For example, PrimeFlow™ (Invitrogen™) or Branched DNA method is a technique to detect cell-expressed RNA by flow cytometry [51], and custom antibody production or customized antibody labeling are also available.

## Conclusion

Here, we presented multiple relatively simple and time-tested approaches that broaden the fluorochrome spectrum for flow cytometric analysis of cells from veterinary relevant species. These approaches can enhance the quality and quantity of information obtained from each cell sample. Therefore, the use of multiparameter analysis in flow cytometry can give critical insight into veterinary and other less commonly analyzed species, can help obtain information from rare cell samples, better define subpopulation of cells, and also potentially reduce the required number of animals used in a study.

## Methods

### Animals and lymphatic cannulation

Eight–eighteen months old female or wether Dorset or Dorset-cross sheep with negative Q-fever serology were purchased from Archer Farms, Inc. and conventionally housed in standard pens, under a 12-h-light/dark cycle, in groups and singly when entering experiments. Hay and water were provided ad libitum, and standard pellet feed for ruminants (Labiana) were fed twice per day. Sheep were 40–65 kg of weight when entering experiments Lymphadenectomy to remove the subiliac (prefemoral) lymph nodes was performed as previously described [52]. Six–eight weeks after lymphadenectomy, pseudoafferent lymph vessels were cannulated with heparin-primed 3 or 3.5 French polyurethane catheters (Access Technologies) in a surgical procedure as described [52]. Pre-procedural sedation was induced with Tilzolan (tiletamine and zolazepam; Dechra) at 4–6 mg/kg into muscles of the hind or front leg; anesthesia was induced with propofol i.v. at 2–8 mg/kg (PropoFlo 28, Zoetis) and/or sevoflurane (Patterson Veterinary) per inhalation at 2–3% in oxygen via mask, and anesthesia was maintained at a surgical plane with 2–3% isoflurane (Isothesia, Covetrus) in oxygen, administered via an endotracheal tube. All surgical procedures were performed under aseptic conditions in a dedicated surgical suite. Postoperative analgesia was provided using buprenorphine (Par Pharmaceuticals) at 0.01–0.05 mg/kg every 4–12 h s.c. in the neck, and/or flunixin meglumine (Flunixin Injection, Norbrook) at 1 mg/kg every 8–24 h i.m. in the leg. Additional doses of analgesics were given if animals showed signs of pain or distress, which were assessed at least three times per day for 3 days, and at least every 12–24 h thereafter for a week. Afferent lymph was collected into sterile bottles containing 100 μL of 10,000 U/mL Heparin (Hospira, Inc.). Collection bottles were changed every 4–12 h. After conclusion of experiments, the animals were euthanized while under anesthesia by i.v. injection with pentobarbital and phenytoin (SomnaSol, Covetrus) at 97.5–195 mg/kg and 12.5–25/kg, respectively. Death was confirmed by auscultation for cardiac arrest. The method of euthanasia is consistent with the recommendations by the Panel of Euthanasia of the American Veterinary Medical Association.

### Cell isolation and blood collection

Blood was collected from the jugular vein with a syringe containing heparin. Mononuclear cells were isolated using density gradient centrifugation with Histopaque®-1083 (Sigma-Aldrich). Blood was diluted at a one to one ratio with elution media (58.4 mM sucrose (Sigma-Aldrich), 10 ml 5 mM EDTA (Invitrogen), 100 mL 10x PBS (Gibco), 900 mL Milli-Q Water) at room temperature and carefully layered on top of the Histopague in a conical tube. The layered blood is centrifuged at 9000 RCF for 30 min. Lymphocytes are collected by harvesting the buffy coat. Blood and lymph-borne cells were washed with wash media (RPMI 1640 medium with GlutaMAX™ (Gibco®), 0.2% BSA (Sigma-Aldrich), and 25 mM HEPES (Gibco®)), and, when necessary, red blood cells were lysed using red blood cell lysing buffer (155 mM ammonium chloride (Sigma-Aldrich), 10 mM sodium bicarbonate (Sigma-Aldrich), and 0.1 mM EDTA (Gibco)). Isolated cells were resuspended in wash media, counted by hemocytometer, and kept on ice until staining.

### Flow cytometry

Mouse monoclonal anti-ovine antibodies recognizing the following ovine antigens were used in the study: CD4 (clone 44.38; BioRad), CD8 (38.65; BioRad), γδ TCR (86D; Washington State University Monoclonal Antibody Center), CD62L (DU1–29; Washington State University Monoclonal Antibody Center), and NKp46 (EC1.1; Timothy Connelly and Lindert Benedictus, The Roslin Institute); CD11c (17–196) and pan-B cell marker (2–104) were kindly provided as hybridoma supernatant or purified antibody by Alan Young (South Dakota State University) and Isabelle Schwartz-Cornil (Institut national de la recherche agronomique). The following mouse monoclonal antibodies, cross-reactive with sheep antigens [39, 40], were used: α4-integrin (HP2/1; Novus Biologicals), β1-integrin (TS2/16; eBioscience), and CD21 (CC21; BioRad). The following secondary antibodies were employed in our study: rat anti-mouse IgG_1_ BUV395 (A85–1, BD Biosciences), rat anti-mouse IgG_1_ PE-Cy7 (RMG1–1; Biolegend), rat anti-mouse IgM (II/41, BD Biosciences), and mouse anti-human IgG_1_ APC (97,924; R&D Systems). Streptavidin-conjugated PerCP-Cy5.5 (BD Bioscience) was used as a secondary or tertiary step reagent. Unlabeled antibodies were labeled with Zenon™ kits or Molecular Probes (Invitrogen™) antibody/protein conjugation kits, as indicated in each Figure, following the manufacturer’s instructions (Invitrogen™, ThermoFisher Scientific). The Zenon™ labeling blocking step was performed with purified whole mouse IgG (Jackson ImmunoResearch).

All staining steps were performed in a total volume of 100 μl on ice. Cells were washed with staining buffer (Dulbecco’s phosphate-buffered saline (DPBS; Corning) and 0.2% tissue-culture grade bovine serum albumin (BSA; Sigma-Aldrich)) and spun down at 500 RCF. 2 × 10^6^ cells were stained per 1.2 mL microtiter tube (Fisher Scientific). To block nonspecific binding, each tube of cells was resuspended, and subsequently incubated for 10 min, in 10 μl of staining buffer containing 1 μg sheep IgG (Jackson ImmunoResearch) and/or 1 μg mouse IgG (Jackson ImmunoResearch), as well as 0.1 μl of the LIVE/DEAD™ Fixable Aqua Dead Cell Stain Kit (Invitrogen™). The blocking step was performed at the beginning of the staining process or after staining with a secondary antibody before addition of antibodies of the same isotype as the primary antibody (Fig. 2 and Fig. 4 a and b). After blocking, antibodies to cell surface antigens were added and cells incubated for 15 min, and subsequently washed with staining buffer. Cells were then fixed by resuspending and incubating for 15 min in 2% paraformaldehyde (Sigma Aldrich), followed by washing with staining buffer. After fixation, ovine CD3 staining was performed in staining buffer containing 0.5% saponin (from *Quillaja* bark, molecular biology grade; Sigma Aldrich). Binding to a mouse E-selectin-human IgG_1_ chimeric protein (R&D Systems) was tested in DPBS containing calcium and magnesium (Corning), and visualized by an APC-conjugated mouse anti-human IgG_1_ antibody (clone 97,924; R&D Systems). The control was stained in the same manner using DPBS without calcium and magnesium under the addition of 30 mM ethylenediamine tetraacetic acid (EDTA; Invitrogen™). Data was acquired using the BD LSR Fortessa™ (BD Biosciences) and analyzed with FlowJo software (Tree Star). A single cell gate was set for each cell sample using FSC-Area and FSC-Height as depicted in Fig. 2a. Dead cells were excluded from analysis by gating on LIVE/DEAD™ Fixable Aqua^low^ events, and lymphocyte and/or granulocyte gates were drawn based on SSC-Area and FSC-Area (Fig. 2a). A minimum of 100,000 lymphocytes were recorded per tube*.*

## Data Availability

The datasets used and/or analyzed during the current study are available from the corresponding author on reasonable request.

## Abbreviations

AF™: Alexa Fluor™
APC: Allophycocyanin
BSA: Bovine serum albumin
BV: Brilliant Violet
DPBS: Dulbecco’s phosphate-buffered saline
EDTA: Ethylenediamine tetraacetic acid
FACS: Fluorescence activated cell sorting
Ig: Immunoglobulin
MAb: Monoclonal antibody;
NK cells: Natural killer cells
PBMC: Peripheral blood mononuclear cell
PE: Phycoerythrin
PE-Cy: Phycoerythrin-cyanine
PFA: Paraformaldehyde
RBC: Red blood cell
RCF: Relative centrifugal force
RPMI: Roswell Park Memorial Institute
